# Sustained Release of Gas6 via mPEG-PLGA Nanoparticles Enhances the Therapeutic Effects of MERTK Gene Therapy in RCS Rats

**DOI:** 10.3389/fmed.2021.794299

**Published:** 2021-12-14

**Authors:** Shen Wu, Yingyan Mao, Qian Liu, Xuejing Yan, Jingxue Zhang, Ningli Wang

**Affiliations:** ^1^Beijing Institute of Ophthalmology, Beijing Tongren Eye Center, Beijing Tongren Hospital, Capital Medical University, Beijing Ophthalmology & Visual Sciences Key Laboratory, Beijing, China; ^2^Collaborative Innovation Center for Brain Disorders, Beijing Institute of Brain Disorders, Capital Medical University, Beijing, China; ^3^Beijing Advanced Innovation Center for Big Data-Based Precision Medicine, Beijing Tongren Hospital, Beihang University, Capital Medical University, Beijing, China

**Keywords:** retinitis pigmentosa, Gas6 nanoparticles, sustained release, phagocytosis, gene therapy

## Abstract

Previous researches utilizing MER proto-oncogene tyrosine kinase (MERTK) gene therapy in Royal College of Surgeons (RCS) rats evidenced its effectiveness in treating MERTK-associated retinitis pigmentosa (RP). Specific ligands for receptor tyrosine kinases, such as growth arrest-specific 6 (Gas6), may enhance retinal phagocytosis *via* the MERTK receptor, and consequently, enhance the therapeutic effects of gene therapy. In order to overcome the short life effect of the injected Gas6 protein, we constructed a Gas6 loaded methoxy-poly (ethylene glyeol)-poly (lactic-co-glycolic acid) (mPEG-PLGA) nanoparticles (Gas6 NPs) system which allowed for localized and sustained Gas6 protein release, and therefore, a prolonged biological effect. Our data demonstrated that Gas6 protein release from Gas6 NPs preserved the bioactivity and promoted retinal pigment epithelium (RPE) phagocytosis *in vitro*. *In vivo* studies showed that RCS rats in the hMERTK/Gas6 NPs group exhibiting the highest electroretinogram responses and more complete retinal structure than that in other groups, further demonstrating that the co-administration of AAV2-BEST1-hMERTK and Gas6 NPs could protect photoreceptors from degeneration. These findings strongly suggest that Gas6 NPs are a promising method to enable the sustained release of Gas6 protein and could therefore enhance the therapeutic effects of gene therapy for MERTK-associated RP.

## Introduction

Retinitis pigmentosa (RP), a group of progressive, hereditary diseases that causes irreversible vision loss, is responsible for blindness in more than 2 million people worldwide (1, 2). Mutations in more than 70 genes have been associated with RP (1). One of the mutation genes is the MER proto-oncogene tyrosine kinase (MERTK), which encodes a transmembrane receptor tyrosine kinase, have been identified to cause RP in patients (3). Two large-scale molecular surveys of retinal dystrophies revealed ~3% of RP cases are attributable to MERTK mutations (4, 5). Such mutations result in defective phagocytosis, which causes the retinal pigment epithelium (RPE) failing to shed photoreceptor outer segments (6).

Numerous studies have reported on the effectiveness of gene replacement therapy for MERTK-associated RP (7, 8). For example, by transplanting an RPE-specific AAV vector, AAV-VMD2-hMerTK, into subretinal space or vitreous cavity could provide long-term photoreceptor rescue in the RCS rats (MERTK-associated retinal dystrophy model) (9, 10). However, although pre-clinical animal models and initial clinical trials suggested a beneficial effect, double-blinded clinical trials with large cohorts of patients failed to show efficacy (11). These disappointing results were attributed, at least partially, long-term MerTK mutations can lead to destruction of retina microenvironment (12). Clearly, it is important to explore more effective therapies to rescue retinal function and morphology in individuals with MERTK-associated RP, especially after the initiation of retinal degeneration.

Specific ligands for receptor tyrosine kinases, such as growth arrest-specific 6 (Gas6), may enhance retinal phagocytosis *via* the MERTK receptor (13). Modulating local environmental factors by Gas6 to provide conditions that are more conducive for functional rescue and repair may maximize the therapeutic effect for RP due to phagocytic dysfunction. However, using Gas6 protein as a drug has many disadvantages, such as short half-life and chemical instability *in vivo*, and may necessitate frequent intraocular injections to maintain long-term effects. This can lead to many complications, such as inflammation, bleeding, and patient compliance issues, which will greatly limit its widespread practical application. To overcome these drawbacks, sustained-release formulations that deliver the protein continuously, thus maintaining the concentration within the therapeutic window for an extended period, have been explored (14). Encapsulating proteins into injectable microspheres or nanoparticles comprised of biodegradable polymers ensures that it maintains its properties and activities (15, 16). Polymers derived from D,L-lactic and glycolic acids, like poly(lactide-co-glycolide) (PLGA), are widely employed with the latter aim in mind (17, 18). PLGA has been approved for use in drug and protein delivery systems by the United States Food and Drug Administration (U.S. FDA) due to its controlled and sustained-release properties, low toxicity, and biocompatibility with tissue and cells.

**Figure 1:**
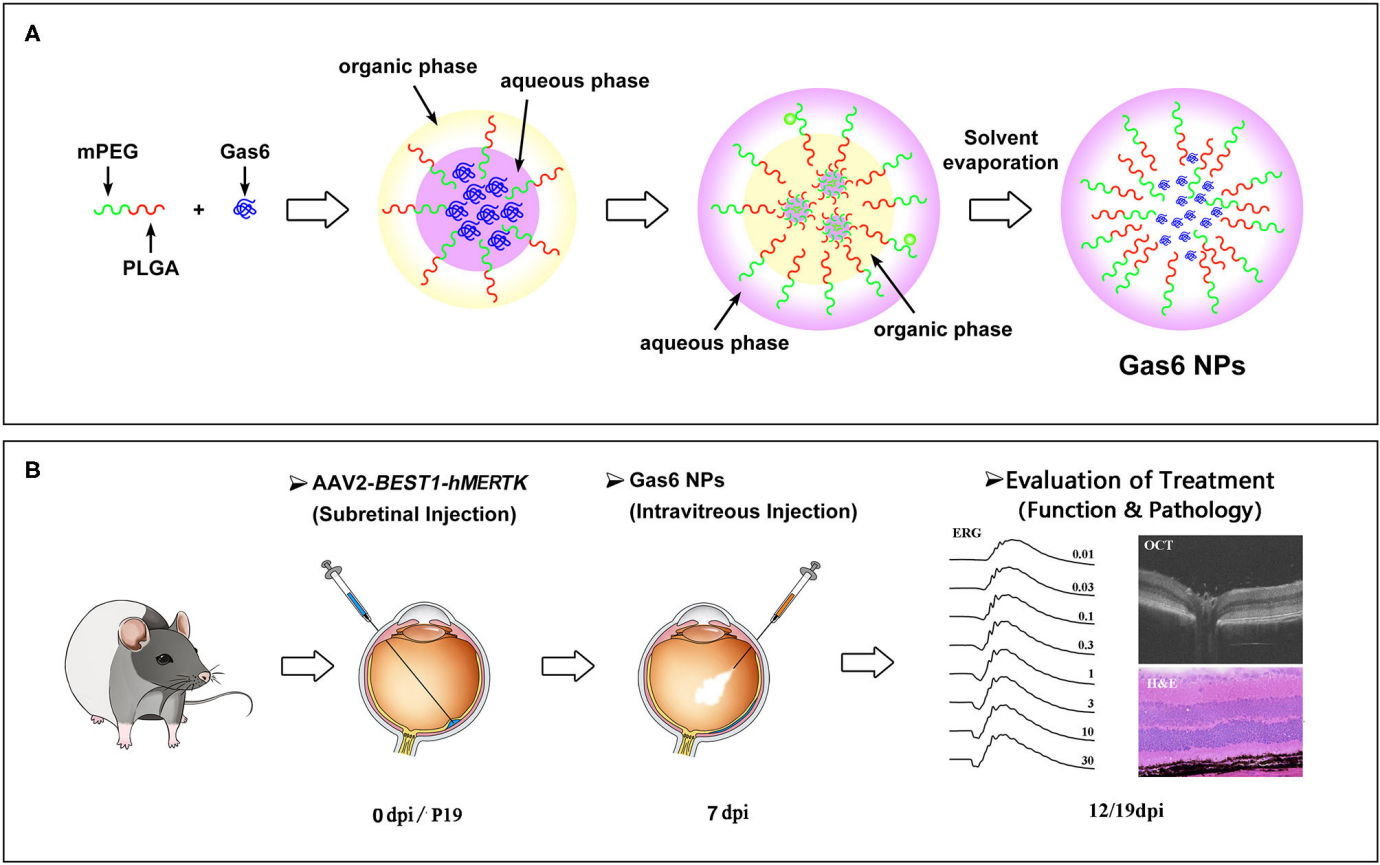
Schematic illustration of **(A)** the preparation of Gas6 NPs and **(B)** Evaluation of the protect effects by co-administration of AAV2-BEST1-hMERTK and Gas6 NPs in RCS rats. dpi: days post injection.

In this study, Gas6 protein was encapsulated into methoxy-poly (ethylene glyeol)-poly (lactic-co-glycolic acid) (mPEG-PLGA) nanoparticles (Gas6 NPs) using the double emulsion technique (Figure 1A). We investigated the bioactivity of Gas6 protein released from Gas6 NPs *in vitro*. To assess whether Gas6 NPs enhance the therapeutic effects of gene therapy for MERTK-associated RP *in vivo*, we co-transplanted AAV2-BEST1-hMERTK and Gas6 NPs (hMERTK/Gas6 NPs) into RCS rats to demonstrate its therapeutic potential in terms of visual function (Figure 1B). In addition, we examined whether the AAV2-BEST1-hMERTK/Gas6 NPs system is effective in rescuing photoreceptors from degeneration in RCS rats.

## Results

### Characterization and *in vitro* Protein Release of Gas6 NPs

**Figure 2:**
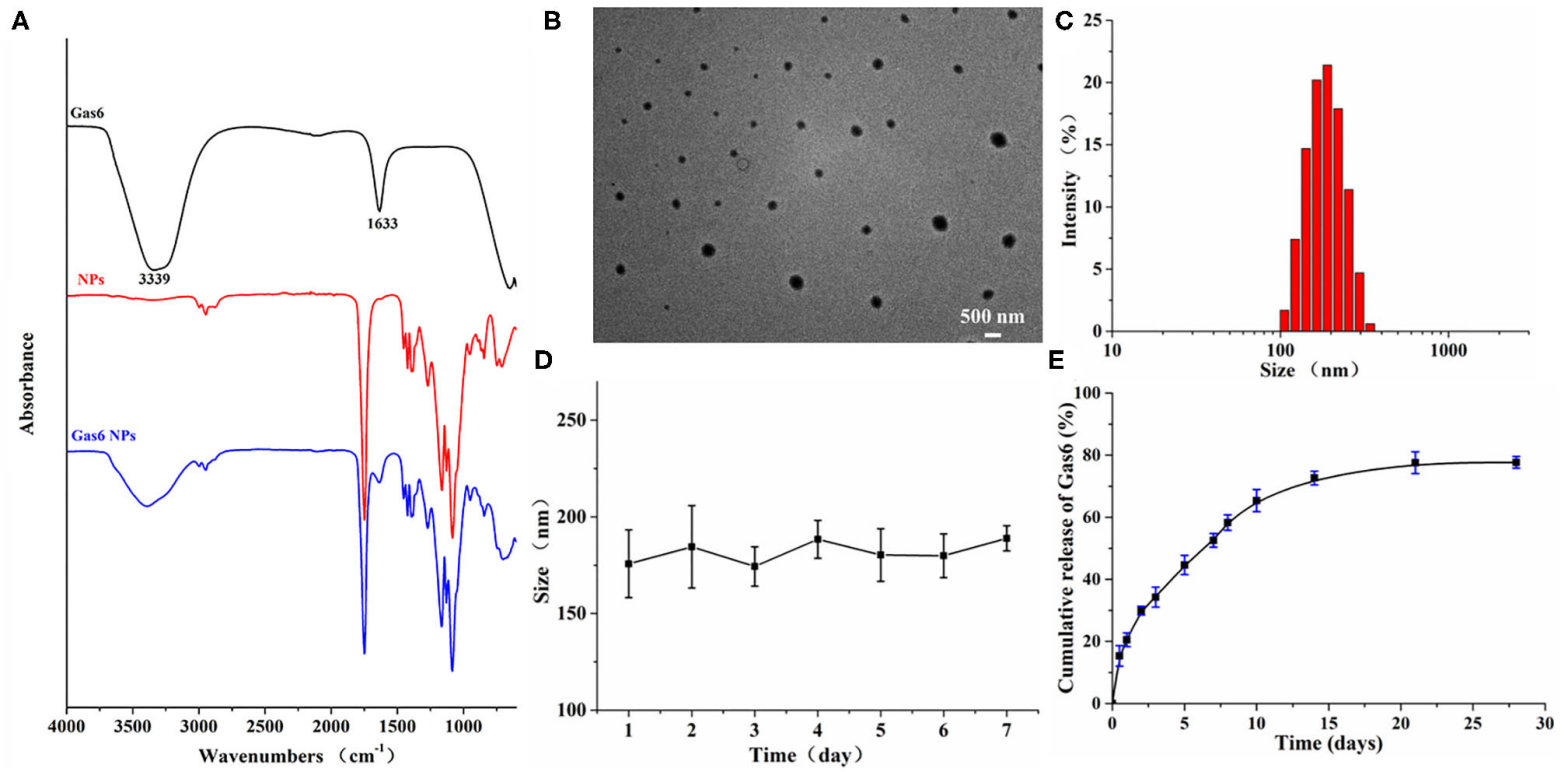
Characterization of Gas6 NPs. **(A)** FTIR spectra of Gas6, NPs, and Gas6 NPs. **(B)** TEM image of Gas6 NPs. Scale bar=200 nm. **(C)** DLS characterization of Gas6 NPs. **(D)** Stability of Gas6 NPs sizes during 7 days characterized by DLS (*n* = 3). **(E)** Cumulative Gas6 release of Gas6 NPs (*n* = 3) at 37°C.

Gas6 NPs were prepared using the double emulsion (w/o/w) technique as illustrated in Figure 1A. The encapsulation of Gas6 into mPEG-PLGA nanoparticles was confirmed by FTIR spectroscopy (Figure 2A). In the FTIR spectrum of Gas6, the peaks at 1,633 and 3,339 cm^−1^ are attributed to N-H deformation and C=O stretching vibrations, respectively. Compared to the FTIR spectrum of pure mPEG-PLGA nanoparticles (blank NPs), the peaks at 1,633 and 3,339 cm^−1^ corresponding to the signal of Gas6 appeared in the FTIR spectrum of Gas6 NPs, confirming the presence of Gas6 in Gas6 NPs.

The encapsulation percentage of the resultant Gas6 NPs was 75%. A TEM image of the resultant Gas6 NPs is presented in Figure 2B. It is observed that Gas6 NPs have a well-defined spherical morphology and high uniformity. The average hydrodynamic diameter of Gas6 NPs determined by DLS was 175.3 nm (Figure 2C). DLS data also evidenced low polydispersity and asymmetric size distributions. As observed by DLS, the size of Gas6 NPs in room temperature water can be maintained at ~180 nm for at least 7 days (Figure 2D). Combining the results of FTIR spectroscopy, TEM observations, and DLS characterizations, it can be concluded that Gas6 was successfully encapsulated into Gas6 NPs.

Gas6 protein release kinetics were determined by the Human Gas6 Elisa Kit (19). Based on the standard curve established in advance, the cumulative amount of Gas6 released at each time point was calculated. The Gas6 release profile exhibited a gradual sustained release pattern throughout the experimental period (Figure 2E) and indicated that Gas6 can be continuously released for more than 2 weeks.

### *In vitro* Biocompatibility and Bioactivity of Gas6 NPs

**Figure 3:**
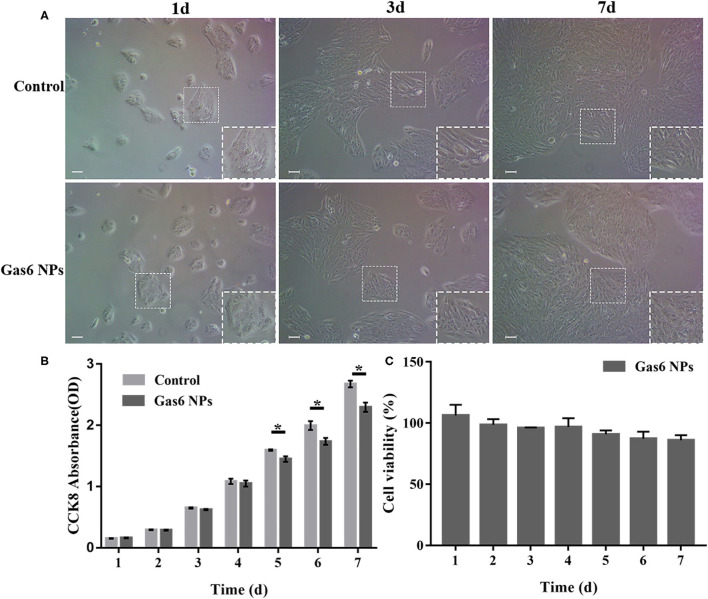
Results of the *in vitro* biocompatibility and cytotoxicity. **(A)** Microscope images of hfRPE cells cultured for 1, 3, and 7 d. **(B)** CCK-8 OD450 of hfRPE cells co-cultured with/without Gas6 NPs during 7 days. **(C)** Cell viability of hfRPE cells co-cultured with Gas6 NPs during 7 days. The data are expressed as mean ± SD (All tests were performed in triplicate, bar = 50 μm). *P < 0.05.

In order to be clinically applicable, a material must have excellent biocompatibility. We therefore investigated the effect of Gas6 NPs on the growth and proliferation of hfRPE cells to determine its biocompatibility. As depicted in Figure 3A, the hfRPE cells co-cultured with Gas6 NPs exhibited similar cell morphology and proliferation as the control group. Moreover, *in vitro* biocompatibility of Gas6 NPs was assessed using CCK8 (Figure 3B). The results demonstrated that the absorbance of CCK8 in the group treated with Gas6 NPs was almost the same as that of the control group before 4 d, but was slightly lower than that of the control group from 5 d to 7 d. We then calculated the relative proliferation rate, which represents cell viability. As indicated in Figure 3C, the cell viability of Gas6 NPs decreases from day 1 to 6 and stabilizes on day 7. The cell viability is 87.61 ± 1.23% on day 7 in the group treated with Gas6 NPs. According to the evaluation criterion in the International Standard ISO10993, the cytotoxicity of Gas6 NPs can be given a ranking of 1 and qualified. This result demonstrated that the Gas6 NPs system has good biocompatibility.

**Figure 4:**
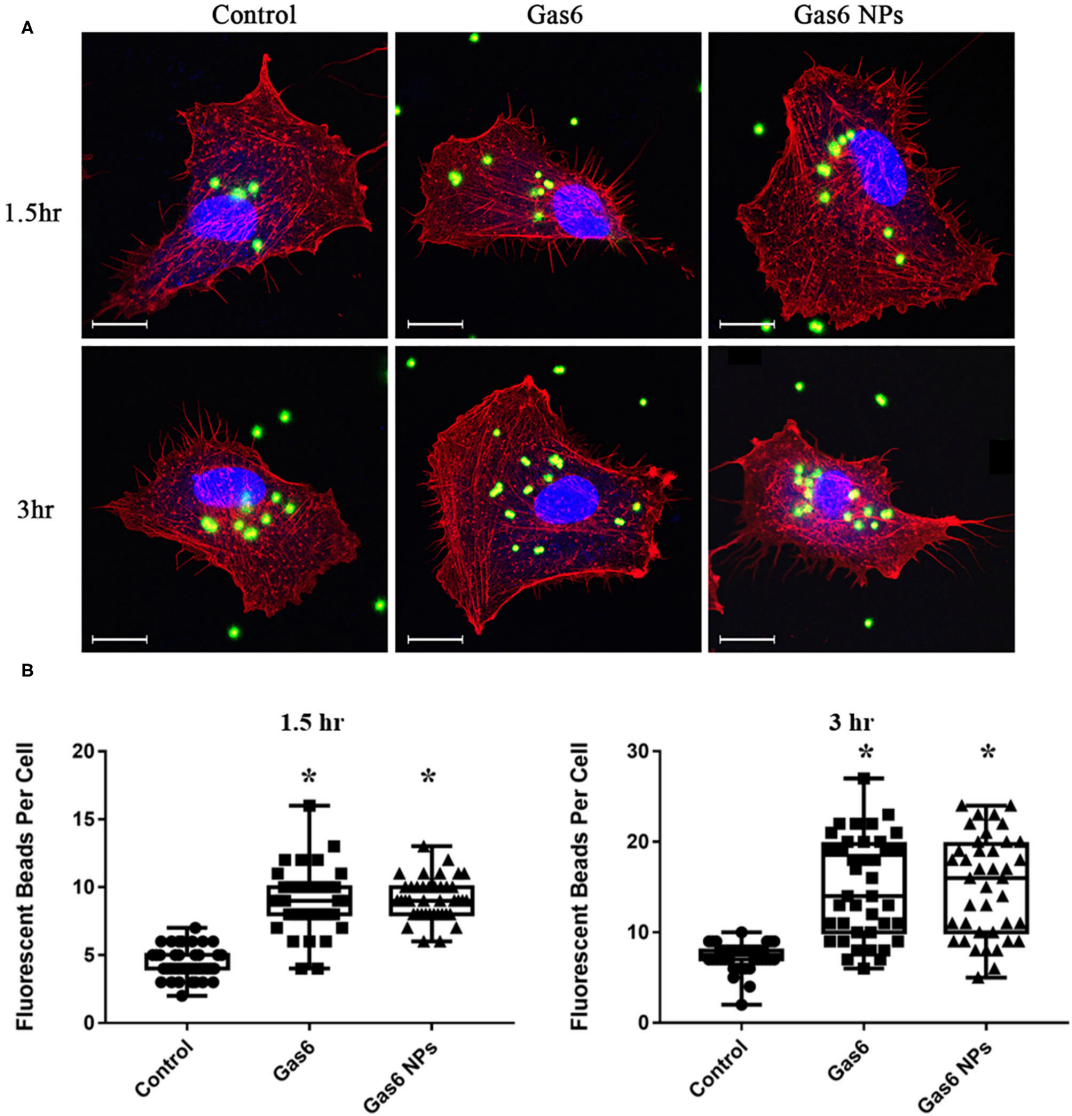
Phagocytosis Assay of human fRPE cells pretreating with Gas6 and Gas6 NPs at 1.5 and 3 h. **(A)** Laser Confocal Microscope images of hfRPE cells pretreating with Gas6 and Gas6 NPs at 1.5 and 3 h. **(B)** Average number of fluorescent beads engulfed by single hfRPE cell. The data are expressed as mean ± SD (tests were performed in triplicate and 40 cells were counted in all, bar = 25 μm). *P < 0.05.

Thereafter, we examined the effects of Gas6 and Gas6 NPs on the phagocytic function of hfRPE cells by phagocytosis assay. When pretreating with Gas6 protein and Gas6 NPs, the location of the fluorescent beads engulfed by hfRPE cells was observed by laser confocal microscope (Figure 4A). The number of fluorescent beads engulfed by hfRPE cells in both Gas6 and Gas6/NPs groups were significantly higher than that of the control group at 1.5 h (8.95 ± 2.30 and 9.15 ± 1.51, respectively, compared to 4.43 ± 1.13 engulfed beads/cell) and 3 h (14.88 ± 5.54 and 15.08 ± 5.51, respectively, compared to 7.33 ± 1.47 engulfed beads/cell) (Figure 4B).

**Figure 5:**
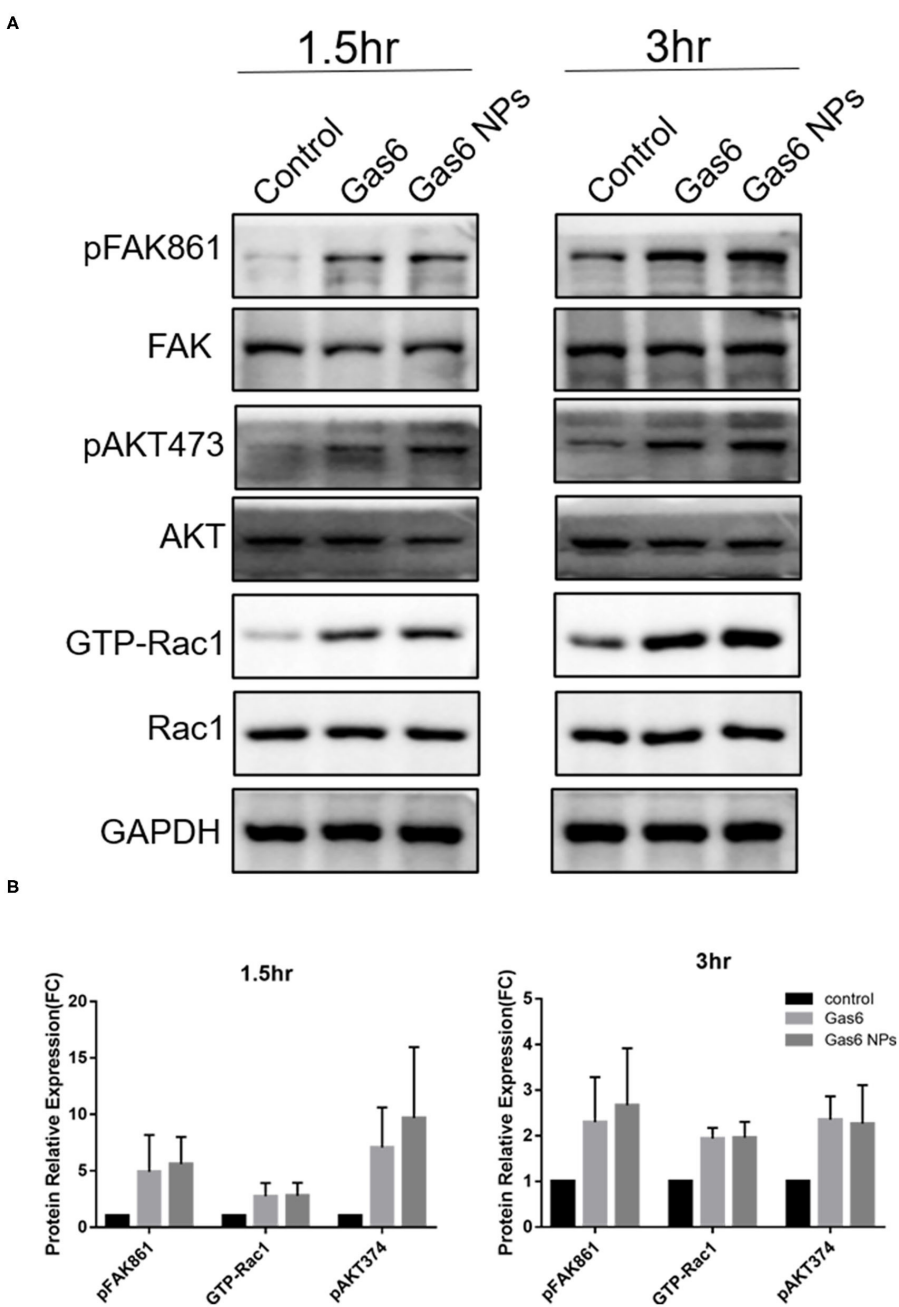
Western blot analysis of the key proteins involved in Gas6 induced phagocytosis. **(A)** Relative optical density determined by densitometry using ImageJ software. **(B)** Protein relative expression in control, Gas6 and Gas6 NPs groups. The data are expressed as mean ± SD.

The Gas6 protein is the ligand of the MERTK receptor and can enhance phagocytosis by RPE cells *via* the MERTK-FakY861-Rac1 signaling pathway (13). Therefore, we detected key proteins involved in Gas6-induced phagocytosis by western blotting (Figure 5A). The fold changes of pFAKY861, GTP-Rac1, and pAKT473 in hfRPE cells pretreated with Gas6 (4.911 ± 3.262, 2.744 ± 1.181, and 7.061 ± 3.547) and Gas6 NPs (5.591± 2.412, 2.784 ± 1.159, and 9.680± 6.285) were higher than that of the control group 1.5 h after the beads were added (Figure 5B). When the incubation time was extended to 3 h, the expression of active proteins remained higher than that of the control group and fold changes were computed as 2.300 ± 0.985 (pFAK861), 1.939 ± 0.232 (GTP-Rac1), and 2.352 ± 0.513 (pAKT473) pretreated with Gas6 and 2.667 ± 1.250 (pFAK861), 1.957 ± 0.348 (GTP-Rac1), and 2.266 ± 0.845 (pAKT473) pretreated with Gas6 NPs (Figure 5B). Collectively, these results suggest that Gas6 NPs provide a safe and effective mean for delivery of Gas6 *in vitro*.

### *In vivo* Rescue of MERTK-Associated Retinal Degeneration by Gas6 NPs

**Figure 6:**
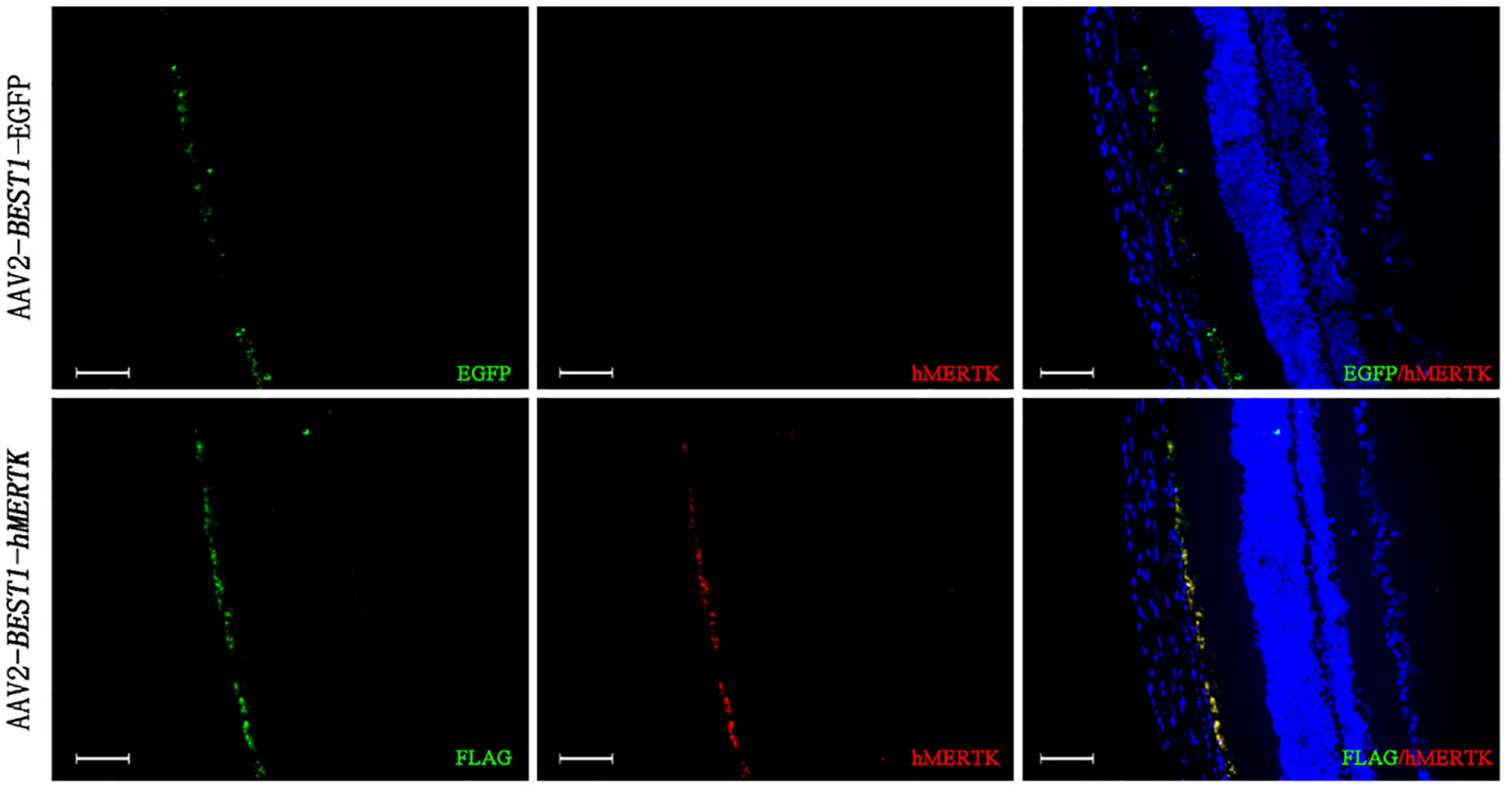
Detection of MERTK expression in RCS rats' retinas following delivery of AAV2-BEST1-hMERTK. hMERTK (labeled as red) expression by immunofluorescence (bar = 50 μm).

RCS rats with inherited retinal degeneration caused by a deletion in the *MERTK* gene were used to evaluate the protective effect of the combination strategy. Firstly, we demonstrated that the hMERTK protein could be sexpressed in the RPE cell layer of RCS rats (Figure 6) following the subretinal injection of AAV2-*BEST1*-hMERTK.

**Figure 7:**
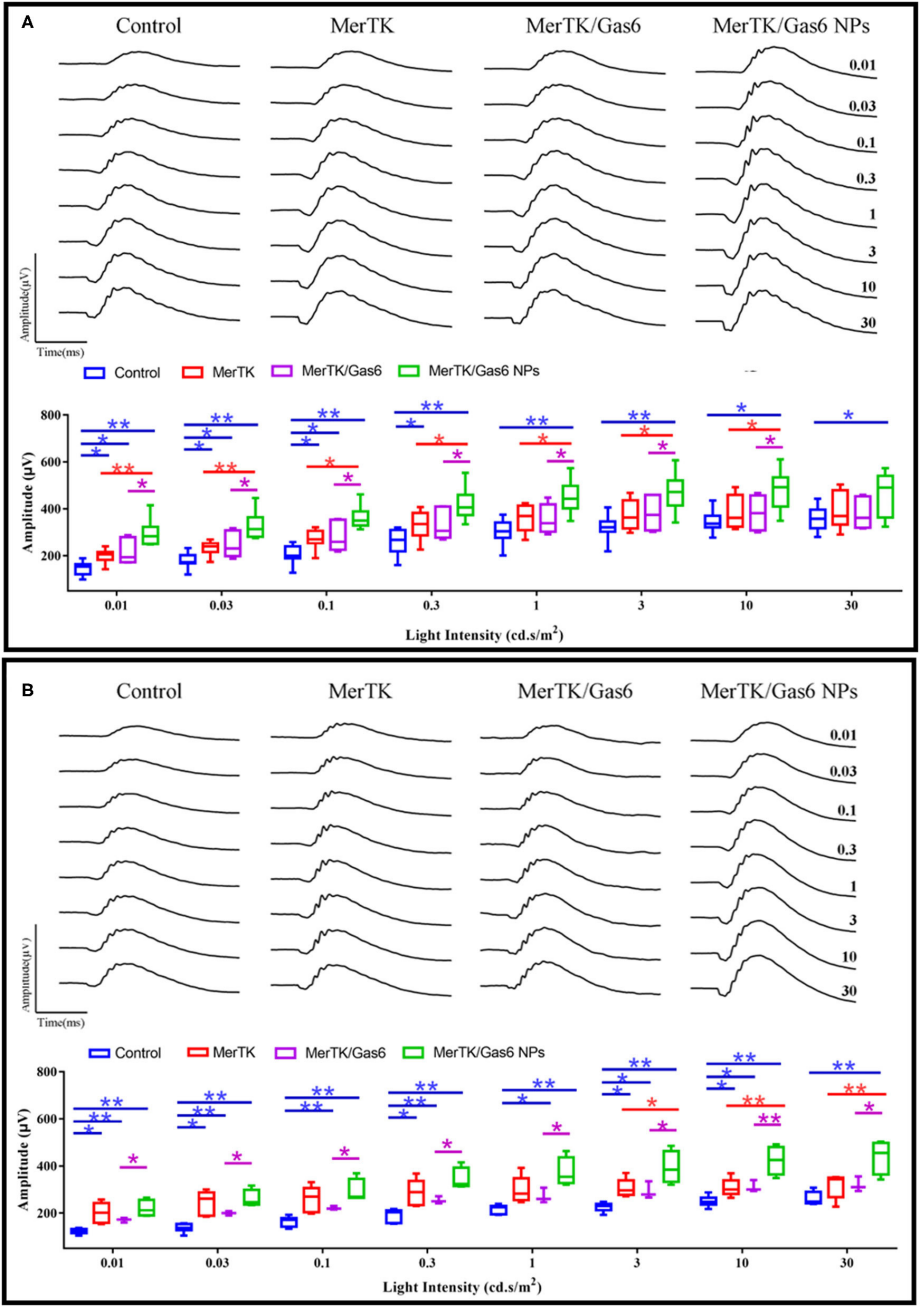
B-wave of dark-adapted (scotopic) ERG data of the representative RCS rats at 12 dpi and 19 dpi. **(A)** Scotopic ERG response (12 dpi) at a series of intensity from 0.01 to 30 cd*s/m2. Control: *n* = 7; MerTK: *n* = 9; MerTK/Gas6: *n* = 7; MerTK/Gas6 NPs: *n* = 6**. (B)** Scotopic ERG response (19 dpi) at a series of intensity from 0.01 to 30 cd*s/m2. Control: *n* = 6; MerTK: *n* = 5; MerTK/Gas6: *n* = 3; MerTK/Gas6 NPs: *n* = 4. *P < 0.05, **P < 0.005.

Additionally, in order to assess whether Gas6 NPs enhanced the therapeutic effects of gene therapy in RCS rats, we recorded and analyzed the ERG response and OCT results of the rats at 12 days post injection (12 dpi, 5 days after Gas6 and Gas6 NPs intervention) and 19 dpi (12 days after Gas6 and Gas6 NPs intervention). The RCS rats in all groups demonstrated typical ERG responses with a and b-waves (Figure 7 and Supplementary Figure 1). At 12 dpi, the hMERTK/Gas6 NPs group exhibited significantly higher b-wave (295.533 ± 61.598 μV) in dark-adapted ERG responses at 0.01 cd.s/m^2^ than other groups. The b-wave amplitudes of the hMERTK group (199.022 ± 28.187 μV) and hMERTK/Gas6 group (214.114 ± 50.677 μV) were also higher than that of the control group (146.179 ± 29.720 μV) at 0.01 cd.s/m^2^; there is no significant difference between the hMERTK group and hMERTK/Gas6 group (Figure 7A).To evaluate the sustained protective effect of the combined treatment, we also recorded ERGs at 19 dpi. As illustrated in Figure 7B, the dark-adapted ERG responses in the hMERTK, hMERTK/Gas6 and hMERTK/Gas6 NPs groups were all significantly higher than that of the control group at low light intensity (0.01 cd.s/m^2^). Moreover, the hMERTK/Gas6 NPs group had the largest b-wave amplitude among the 3 treatment groups, similar to that recorded at 12 dpi. There was no significant difference between the hMERTK and hMERTK/Gas6 groups at 19 dpi too.

**Figure 8:**
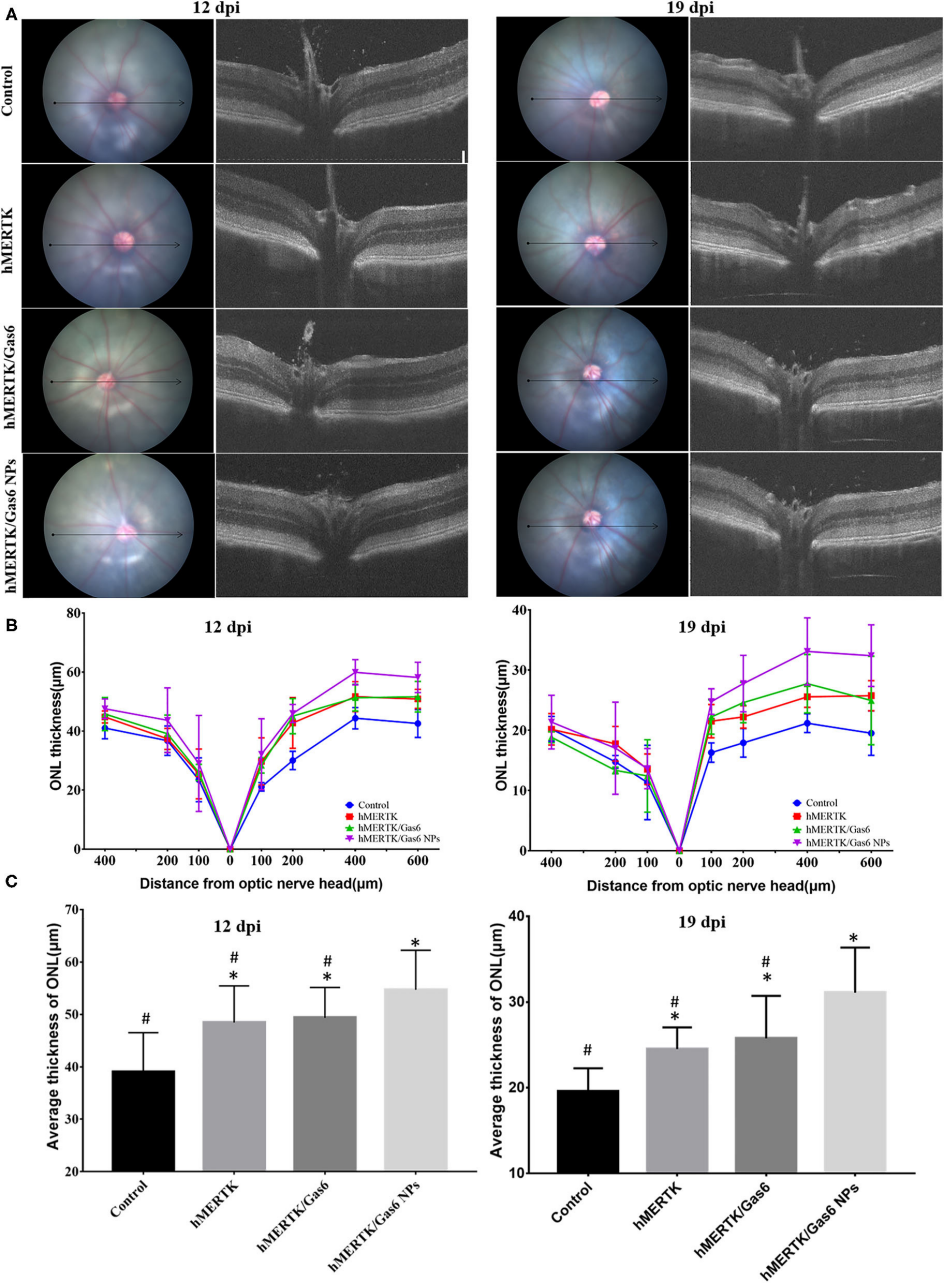
Architecture in the retina of the representative RCS rats at 12 dpi and 19 dpi. **(A)** Representative retinal cross section B-scan OCT image (the location was indicated with a black line in ocular fundus) at 12 dpi and 19 dpi; **(B)** Quantitative measurements of retinal thickness in different locations at 12 dpi and 19 dpi; **(C)** Average thickness of temporal retina at 200, 400, and 600 microns at 12 dpi and 19 dpi. *P < 0.05 vs. control group; ^#^*P* < 0.05 vs. hMERTK/Gas6 NPs group. Sample size at 12 dpi: Control: *n* = 6; MerTK: *n* = 5; MerTK/Gas6: *n* = 5; MerTK/Gas6 NPs: *n* = 6. Sample size at 19 dpi: Control: *n* = 3; MerTK: *n* = 4; MerTK/Gas6: *n* = 3; MerTK/Gas6 NPs: *n* = 4.

We further evaluated the architecture in the retina of the RCS rats *via* OCT. Representative OCT images of the retina were taken horizontally across the optic nerve head (ONH), and the imaging location was marked on the fundus image with a black line (Figure 8A). Then we quantitatively measure the thickness of the outer nuclear layer (ONL) at 100, 200, 400-μm of temporal retina and 100, 200, 400, 600-μm of nasal retina from the ONH (Figure 8B). We can clearly note that the ONL thickness curve of the nasal side was clearly separated (Figure 8B). The average ONL thickness of the nasal retina was calculated and shown in Figure 8C. After co-administration of AAV2-BEST1-hMERTK and Gas6 NPs, the thickness of nasal ONL was 54.70 ± 7.53 μm and 31.09 ± 5.27 μm respectively at 12 dpi and 19 dpi. The thickness of the ONL was significantly higher than that of the Control group (39.00 ±7.50 μm and 19.54 ± 2.73 μm), hMERTK group (48.45 ± 6.98 μm and 24.51 ± 2.52 μm,) and hMERTK/Gas6 group (49.35 ± 5.78 μm and 25.77 ± 4.95 μm,).

**Figure 9:**
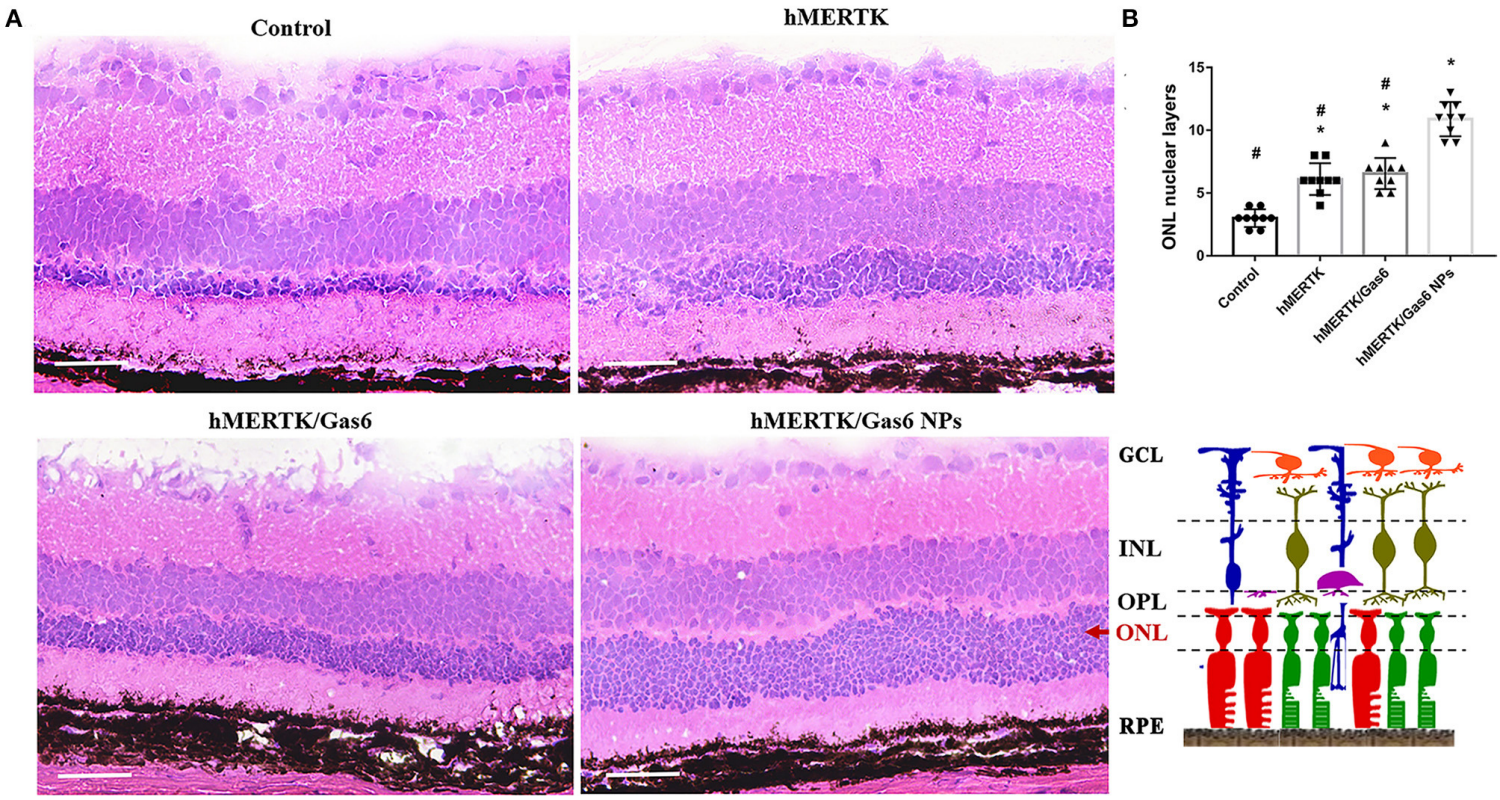
Histologic structure of RCS rats at 19 dpi. **(A)** Hematoxylin–eosin (H&E) staining of retina. **(B)** The ONL nuclear layers of the retina. The data are expressed as mean ± SD. *P < 0.05 vs. control group; ^#^*P* < 0.05 vs. hMERTK/Gas6 NPs group. GCL, Ganglion cell layer; IPL, Inner plexiform layer; INL, Inner nuclear layer; OPL, Outer plexiform layer; ONL, Outer nuclear layer; RPE, Retinal pigment epithelium. *P < 0.05, bar, 50 μm.

The rats were finally sacrificed and the eyeballs were taken out. The H&E staining images of retina were shown in Figure 9A. The results evidenced that the ONL nuclear layers in control group (3 ± 0.71) was significantly less than that of the hMERTK group (6.11 ± 1.23), hMERTK/Gas6 group (6.56 ± 1.24), and hMERTK/Gas6 NPs group (10.89 ± 1.36) (Figure 9B). A statistically significant difference was observed between the hMERTK/Gas6 NP group and the other 2 treatment groups. However, there was no significant difference between the hMERTK and hMERTK/Gas6 group. And the result of the ONL thickness is consistent with the result of ONL nuclear layers (Supplementary Figure 2). Altogether, these results suggest that the combined treatment of AAV2-BEST1-hMERTK and Gas6 NPs had a greater protective effect on photoreceptors.

## Discussion

In this study, we prepared Gas6 protein-loaded mPEG-PLGA nanoparticles (Gas6 NPs), which allowed for localized and sustained Gas6 protein release to overcome the short half-life of the Gas6 protein *in vivo*. We identified that Gas6 NPs preserved Gas6 protein bioactivity and promoted RPE phagocytosis *in vitro*. Additionally, we assessed whether Gas6 NPs enhanced the therapeutic effects of gene therapy in RCS rat model of MERTK-associated retinal dystrophy. Our results demonstrate that the co-administration of AAV2-BEST1-hMERTK and Gas6 NPs could protect photoreceptors from degeneration in RCS rats. Consequently, the ERG response was remarkably ameliorated and more structure of retina was preserved in the hMERTK/Gas6 NPs group. These findings strongly suggest that Gas6 NPs are a promising method for the sustained release of Gas6 protein and could enhance the therapeutic effect of gene therapy for MERTK-associated RP.

MERTK gene replacement therapy is considered as a promising treatment for MERTK-associated RP and demonstrated therapeutic efficacy (20–23). Recent studies have indicated that the use of Gas6 enhances retinal phagocytosis *via* the MERTK receptor either alone or in combination with other specific ligands for receptor tyrosine kinases, which may enhance the therapeutics effects of gene therapy (13). Whereas, Gas6 as a recombinant protein has many disadvantages such as short *in vivo* half-lives and chemical instability, may necessitate frequent administration over time to maintain long term effect. Therefore, we encapsulated Gas6 into mPEG-PLGA nanoparticles (Gas6 NPs) and conducted a series of evaluations to determine the safety and function of Gas6 NPs *in vitro*. Our results demonstrated that both Gas6 protein and Gas6 NPs induced phagocytosis in hfRPE cells, which is consistent with previous reports (13). Moreover, our results confirm the bioactivity of the released Gas6 protein, which provides a solid foundation for the application of Gas6 NPs in enhancing the therapeutic effect of gene therapy.

To confirm the effect of Gas6 NPs *in vivo*, we designed the co-administration strategy of Gas6 NPs with MERTK gene and subsequently evaluated its therapeutic effects in RCS rats. Our results evidenced that the hMERTK/Gas6 group had a similar ERG response to that of the hMERTK group, while the hMERTK/Gas6 NPs group attained the highest amplitude based on ERG response. This suggested the sustained release of Gas6 protein within the therapeutic window for an extended period was essential for enhancing visual function. The mPEG-PLGA formulation would offer numerous advantages, including the protection of Gas6 from degradation or elimination and the ability to deliver Gas6 locally to the retina, it is likely to enhance the therapeutic effect of gene therapy. Moreover, the dark-adapted ERG response of the control, hMERTK, and hMERTK/Gas6 groups was significantly weakened at certain light intensities at 19 dpi compared to 12 dpi, whereas no such difference was observed in the hMERTK/Gas6 NPs group. It is possible that the sustained release of the Gas6 protein induced a prolonged effect within the retinal environment, and consequently enhanced RPE phagocytosis *via* the MERTK receptor for an extended period.

To our knowledge, it is the first time we proposed the concept of sustained release of Gas6 protein within the therapeutic window for enhancing the effects of gene therapy for MERTK-associated RP. Here, the mPEG-PLGA was used to encapsulate Gas6 protein, which has good biocompatible and its degradation depends on molecular weight, conformation, and copolymer composition (24). The Gas6 protein release kinetics obtained in this study demonstrate that mPEG-PLGA nanoparticles are a suitable protein delivery system and, more specifically, an effective sustained-release system. While, nearly 70% of Gas6 had been released from Gas6 NPs by day 12, after which its release slowed down. In the clinical setting, we need the release of Gas6 to be sustained for a longer duration in order to reduce the frequency of administration and increase patient compliance and comfort. This requires us to optimize this sustained-release system in future research to finally achieve an extended-release system.

Our findings strongly suggest that Gas6 NPs could enhance RPE phagocytosis *in vitro* and be used for enhancing the effects of gene therapy for MERTK-associated RP *in vivo*. We propose that the promotion of RPE phagocytosis *via* the sustained released of Gas6 may enhance the therapeutic efficiency of gene therapy in RCS rats. The potential mechanisms underlying the manner in which the Gas6 protein enhances the therapeutic efficiency of MERTK gene therapy is unknown and should be addressed by further studies. However, it has been reported that Gas6 is a multi-functional circulating protein with multiple roles related to inflammation the and immune system (25), and retinitis pigmentosa is usually accompanied by inflammation. Of note, once it is identified that Gas6 enhances the therapeutic effect of MERTK gene therapy by up-regulating phagocytosis and improving the retinal microenvironment, early administration of retinal microenvironment *via* Gas6 sustained release system could be applicable to other subtypes of retinal degenerative diseases.

In summary, we designed the hMERTK/Gas6 NPs co-administration system and evaluated its therapeutic effects on RP treatment. We developed injectable Gas6 NPs with mPEG-PLGA *via* the double emulsion technique. The Gas6 NPs facilitated the development of a localized Gas6 delivery system with improved retention time. *In vitro* studies demonstrated that the Gas6 NPs remarkably increased the phagocytic function of hfRPE cells. Meanwhile, *in vivo* studies evidenced that the co-administration of Gas6 NPs with MERTK gene replacement therapy remarkably ameliorated the functional recovery of the ERG response and preserved more retinal structure.

## Materials and Methods

### Preparation of Gas6 NPs

mPEG-PLGA (mPEG5000-PLGA 75/25 [70000]) was purchased from Shanghai Zhong-Liang Oil and Fat Chemical Co., Ltd. (Shanghai, China). Moreover, polyvinyl alcohol (PVA), pluronic F68, esteramide (EA), and dichloromethane (DCM), all having a purity of at least 99%, were purchased from Sigma Chemical Corp. (St. Louis, Missouri, United States). mPEG-PLGA nanoparticles loaded with Gas6 were prepared using the double emulsion (w/o/w) technique. Briefly, 10 mg mPEG-PLGA was dissolved in 0.5 mL EA, after which 0.1 mL Gas6 solution (3 g/dL) was added. This mixture was transferred to a centrifuge tube and emulsified by sonication for 3 min at 80 W. Thereafter, the resultant emulsion was slowly added to 0.7 mL 2% (m/v) PVA and 0.3 mL 2% (m/v) pluronic F68, which was then stirred vigorously for 10 min. The mixture was subsequently emulsified *via* sonication for 5 min at 250 W. Both emulsification steps were performed in an ice bath. After the solvent was evaporated by applying vacuum, the Gas6 NPs were collected by centrifugation at 10,000 rcf. for 10 min and then washed twice using distilled water.

### Characterization of Gas6 NPs

Fourier transform infrared (FTIR) spectra were recorded using an FTIR spectrometer (Spectrum One, PerkinElmer). The average size of Gas6 NPs was determined by dynamic light scattering (DLS) using a ZetaSizer Nano ZS (Malvern Instruments Ltd., Malvern, Worcestershire, United Kingdom). Samples were appropriately diluted with distilled water, and subsequently measured at 633 nm at 25°C and a constant angle of 90°. Stability experiments were regularly performed for the duration of 1 week by measuring the size of Gas6 NPs in phosphate-buffered saline (PBS) solution at room temperature. The concentration of Gas6 NPs used in the stability experiments was the same as that in the *in vivo* study. Furthermore, the morphology of Gas6 NPs was confirmed using a transmission electron microscope (TEM; JEM-200CX, JEOL Ltd., Tokyo, Japan).

### Determination of the Encapsulation Efficiency

The encapsulation efficiency percentage (EE%) of Gas6 NPs was calculated as follows:


$$\begin{array}{l} EE\%=\frac{{Gas6}_{total}-{Gas6}_{free}}{{Gas6}_{total}}*100\% \end{array}$$

where Gas6_total_ is the total amount of Gas6 in the nanoparticle and suspension, while Gas6_free_ is the amount of free Gas6 in the suspension. Enzyme-linked immune absorbent assay (ELISA) was applied to detect Gas6_free_ in the suspension.

#### *In vitro* Gas6 Release

Gas6 protein release kinetics were determined by the Human Gas6 ELISA Kit (Sigma-Aldrich Corp., St. Louis, Missouri, USA). Briefly, Gas6 NPs were immersed in PBS at 4°C with gentle shaking. At determined intervals, the suspension was centrifuged and the supernatant was replaced and collected. The Gas6 concentration in the supernatant was determined by the Human Gas6 Elisa Kit. The *in vitro* release reactions were carried out in triplicate for each sample.

### Cell Culture and Animals

Human fetal RPE (hfRPE) cells were kindly provided by Professor Guoping Fan(University of California, Los Angeles, California, US) (26). These cells were cultured in DMEM/F12(1:1) medium supplemented with 15% fetal bovine serum (FBS) (SH3007003HI; Thermo Fisher Scientific) at 37°C in a humidified incubator with a 5% CO_2_/95% air atmosphere. The medium was replaced every 2 days.

RCS rats were also kindly provided by Professor Guoping Fan and kept in Capital Medical University,Beijing,China. The animals were kept in temperature-controlled rooms with a 12-h light/dark cycle and were provided with standard food and water *ad libitum*. The study was approved and monitored by the Institutional Animal Care and Use Committee of the Capital Medical University (IACUC; AEEI-2018-198), and conformed to the National Institute of Health Guide for the Care and Use of Laboratory Animals as well as the Association for Research in Vision and Ophthalmology (ARVO) Statement for the Use of Animals in Ophthalmic and Vision Research.

### Cell Viability Assay

The hfRPE cells (0.5 × 10^4^ cells/well) were allowed to adhere overnight after plated in 96-well plates before being incubated for 7 days. Cells were then treated with Gas6 NPs dispersion (final concentration of Gas6 was 500 ng/mL) and incubated for an additional 7 days. Cell viability was tested daily. Briefly, the culture medium was replaced by 200 μL 10% CCK8 reagent (CA1210; Solarbio, Beijing, China) and incubated for 2 h at 37°C. The optical density (OD) value was measured at 450 nm using an ELISA reader (BioTek, Winooski, Vermont, USA) according to the manufacturer's instructions. The relative proliferation rate was calculated as follows: RPR = (OD_TEST_/OD_Control_) ×100%

### Phagocytosis Assay

The hfRPE cells were plated at a density of 5 × 10^4^ cells/well into poly-l-lysine coated 24-well plates and allowed to adhere overnight. Cells were then pretreated with Gas6 NPs dispersion (final concentration of Gas6 was 500 ng/mL) or recombinant Gas6 protein (final concentration 500 ng/mL) for 1 h. Thereafter, 5 μL of 1 μm fluorescein isothiocyanate (FITC)-labeled carboxylate-modified microspheres (1933365; 1:10 dilution; Invitrogen, USA) was added, and initially incubated for 1.5 h and 3 h at 37°C with a 5% CO_2_ atmosphere. The medium was then removed and cells were washed 6 times to remove excess beads. Cells were subsequently labeled with 4′,6-diamidino-2-phenylindole (DAPI) and tetramethylrhodamine (TRITC) phalloidin (CA1610; Solarbio, Beijing, China). Four images were taken of each well using a Leica SP5 microscope. Additionally, the amounts of beads per cell was counted in 40 cells per condition.

### Western Blotting Analysis

The hfRPE cells were plated at a density of 5 × 10^5^ cells/well into poly-l-lysine coated 6-well plates and allowed to adhere overnight. Cells were then pretreated with Gas6 NPs dispersion (final concentration of Gas6 was 500 ng/mL) or recombinant Gas6 protein (final concentration 500 ng/mL) for 1 h. Thereafter, 15 μL of 1 μm fluorescein isothiocyanate (FITC)-labeled carboxylate-modified microspheres (1933365; 1:10 dilution; Invitrogen, USA) was added, and incubated for 1.5 h and 3 h at 37°C with a 5% CO2 atmosphere. Thereafter, proteins were extracted from cells and their total concentration was measured using a BCA Protein Assay Kit (CWBIO) according to the manufacturer's instructions. Equal quantities (40 μg) of proteins per gel lane were separated on 10% polyacrylamide gels by sodium dodecyl sulfate–polyacrylamide gel electrophoresis (SDS-PAGE) and then transferred to polyvinylidene fluoride membranes using an electroblotting apparatus (Bio-Rad). Membranes were blocked using a solution containing 5% non-fat milk and TBS-Tween20 and then incubated separately at 4°C overnight with the following primary antibodies: GAPDH (sc-25778; 1:1000; SantaCruz Biotechnology), active GTP-Rac1 (26903; 1:200; Neweast Biosciences), Rac1 (ab155938; 1:600; Abcam), p-FAK861 (44-626G; 1:800; Invitrogen), FAK (AHO0502; 1:200; Invitrogen), p-AKT473 (4060s; 1:500; CST), and AKT (9272; 1:500; CST). Membranes were then incubated with horseradish-peroxidase (HRP)-conjugated secondary antibody (goat anti-mouse/rabbit IgG antibody; G21240/G21234; 1:1000; Invitrogen) for 1 h at room temperature. Membranes were then washed 3 times (10 min per wash) with 0.1% TBS-Tween20 after each antibody application. Thereafter, immuno-labeled proteins were detected using the ECL Plus Detection System (Invitrogen) according to the manufacturer's instructions. The band was analyzed using Image-Pro Plus (IPP) software.

### Animal Experiments

The pAAV2-*BEST1*-hMERTK vector was prepared by Vigene Biosciences Inc. Briefly, the human MERTK gene and *BEST1* promoter were amplified from a Human Retina Marathon-Ready cDNA Library (Clontech) and cloned into the pUC19 vector. The sequence of the construct was verified by Sanger sequencing. Thereafter, the expression cassette, containing the *BEST1* promoter, hMERTK cDNA, and 3xFLAG tag, was excised by restriction enzymes and cloned into the pAAV plasmid. The pAAV2-*BEST1*-MERTK plasmid was then transfected into HEK293 cells and the virus was harvested. The final concentration of AAV2-*BEST1*-hMERTK was 3.37 × 10^13^ vg/mL. The map of pAAV2-*BEST1*-hMERTK and pAAV2-*BEST1*-EGFP were showed in Supplementary Figure 3. The sequence of BEST1 promoter was list in Supplementary Table 1.

Subretinal injections of the AAV2-*BEST1*-hMERTK virus were administered to RCS rats at P19 (0 dpi). RCS rats were divided into 4 different groups based on the administered treatment, namely Control group (AAV2-*BEST1*-EGFP), hMERTK group (AAV2-*BEST1*-hMERTK), hMERTK/Gas6 group (AAV2-*BEST1*-hMERTK & Gas6 protein), and hMERTK/Gas6 NPs group (AAV2-*BEST1*-hMERTK & Gas6 NPs). Rats were anesthetized using an intraperitoneal injection containing 30 mg/kg pentobarbital sodium and 5 mg xylazine hydrochloride. Pupils were dilated using 0.2 mg/mL tropicamide phenylephrine (Santen Pharmaceutical Co., Ltd., Shiga Plant, Shiga, Japan) and topically anesthetized with 0.5% proparacaine (Santen Pharmaceutical Co., Ltd.). A 31-gauge insulin syringe was used to carefully puncture the temporal corneoscleral limbus. Thereafter, a 33-gauge blunt needle (Hamilton) was used to administer the subretinal injection. The blunt needle tip was inserted through the sclerotic puncture and aimed at the contralateral subretinal space. Subsequently, 1 μL of AAV2-*BEST1*-EGFP (control group) or AAV2-*BEST1*-hMERTK virus (hMERTK, hMERTK/Gas6, and hMERTK/Gas6 NPs groups) was injected into the subretinal space of the RCS rats' right eye. Tobramycin and dexamethasone eye ointment (ALCON) were applied once daily for 3 days after injection.

At 7 dpi, 2 μL PBS (control and hMERTK groups), 2 μL Gas6 protein (hMERTK/Gas6 group; 0.5 μg/uL), or 2 μL Gas6 NPs (hMERTK/Gas6 NPs group; 0.5 μg Gas6/μL) was administered *via* intravitreal injection. The ERG and OCT were recorded at 12 dpi and 19 dpi. Animals exhibiting retinal bleeding and cataracts were excluded.

### Electroretinographic Analysis

The Espion Visual Electrophysiology System (Diagnosys, USA) was used to record the electroretinogram. After at least 12 h of dark adaptation, animals were anesthetized and their pupils were dilated using 0.2 mg/mL tropicamide phenylephrine. Animals were placed on a regulated heating pad throughout the experiment. Electroretinograms (ERGs) were recorded by means of a golden ring that made contact with the corneal surface through a layer of 0.2% carbomer. Additionally, needle electrodes were inserted into the cheeks and tails of animals and served as the reference and ground leads, respectively. Scotopic testing at eight-intensity stimulus-response series (0.01 cd.s/m^2^ to 30 cd.s/m^2^) were presented; the resulting b-wave amplitudes were measured from the trough of the a-wave to the crest of the b-wave.

### Optical Coherence Tomography

OCT was performed using a Micron III (Phoenix Research Labs, Pleasanton, CA). Animals were anesthetized and their pupils were dilated using 0.2 mg/mL tropicamide phenylephrine. The corneal surface was protected using a 1.5% hydroxyethylcellulose solution. The rat ocular fundus was monitored using the fundus camera of the Micron. Representative OCT images of the retina were taken horizontally across the optic nerve head (ONH), and the imaging location was marked on the image with a black line. Thirty images were averaged to eliminate projection artifacts. The acquired OCT images were quantitatively analyzed using the InSight software (Phoenix Research Labs). The thickness of the outer nuclear layer (ONL) was measured at 100, 200, 400-μm of temporal retina and 100, 200, 400, 600-μm of nasal retina from the ONH. The average value of the ONL in nasal retina at 200, 400, and 600 microns was used represent the average thickness of ONL.

### Tissue Immunofluorescence Staining

Samples were cut into 10 μm sections using a cryostat (Leica Microsystems, Wetzlar, Germany). Thereafter, sections were rinsed 3 times with PBS for 5 min at room temperature, and then blocked with 5% bovine serum albumin (BSA) for 30 min at room temperature. Sections were subsequently transferred to a moist chamber containing rabbit recombinant monoclonal anti-hMERTK antibody (ab52968; 1:200; Abcam) at 4°C overnight. Thereafter, sections were rinsed 3 times with PBS and incubated with Alexa Fluor 594 anti-rabbit secondary antibody (R37119; 1:1000; Invitrogen) in the dark for 1 h at room temperature. Cell nuclei were then stained with DAPI and fluorescent signals were visualized using a Zeiss fluorescence microscope (Observer Z1).

### Histological Analysis

To quantify the layers and the thickness of outer nuclear layer (ONL) after combined treatment, hematoxylin and eosin (H&E) staining was performed. Rats were anesthetized using 400 mg/kg chloral hydrate, after which their eyes were enucleated, fixed in 4% paraformaldehyde for 4 h, washed 3 times with PBS, and embedded in paraffin. Eight-micron-thick paraffin sections were used for H&E staining. Stained slices were visualized by a Leica microscope. The ONL layers was counted in a double-blind manner. The thickness of the ONL was measured using Image-Pro Plus (IPP) software. There are 3 animals in each group, and the layers and thickness of ONL of each animal were measured in 3 different slices.

### Statistical Analysis

All data were presented as the mean ± standard deviation (SD). Independent Samples *T*-test analysis was used to compare the CCK8 absorbance between the control group and Gas6 NPs group. One-way analysis of variance (ANOVA) and Fisher's least significant difference (LSD) test were used to determine whether significant differences exist between the ERG amplitudes of the different treatment groups. Two-way analysis of variance and Fisher's least significant difference (LSD) test were used to determine whether significant differences exist among the OCT results. The independent Samples *T*-test analysis was used to compare the differences between the cell viability of each group. Statistical analyses were performed by SPSS 20.0 (SPSS Inc., Chicago, Illinois, USA). *P* < 0.05 indicated statistical significance.

## Data Availability Statement

The original contributions presented in the study are included in the article/Supplementary Material, further inquiries can be directed to the corresponding authors.

## Ethics Statement

The animal study was reviewed and approved by Institutional Animal Care and Use Committee of the Capital Medical University (IACUC; AEEI-2018-198).

## Author Contributions

SW and YM conducted the experiments, analysis the data, and wrote the paper. JZ and NW designed the experiments and revised the paper. QL and XY provided the materials. All authors contributed to manuscript revision, read, and approved the submitted version.

## Funding

This work was supported by National Key R&D Program of China (2017YFA0104100 and 2016YFC0905201), Beijing Municipal Institute of Public Medical Research Development and Reform Pilot Project (2018-2), Beijing Hospitals Authority Youth Program (QML20180208), and Beijing Institute of Ophthalmology Key Program (2019003). These funders had no role in study design, data collection and analysis, decision to publish, or preparation of the manuscript.

## Conflict of Interest

The authors declare that the research was conducted in the absence of any commercial or financial relationships that could be construed as a potential conflict of interest.

## Publisher's Note

All claims expressed in this article are solely those of the authors and do not necessarily represent those of their affiliated organizations, or those of the publisher, the editors and the reviewers. Any product that may be evaluated in this article, or claim that may be made by its manufacturer, is not guaranteed or endorsed by the publisher.

## References

[1] Dias MF Joo K Kemp JA Fialho SL Jessica A Woo SJ. Molecular genetics and emerging therapies for retinitis pigmentosa: basic research and clinical perspectives. Prog Rtein Eye Res. (2018) 63:107–31. 10.1016/j.preteyeres.2017.10.004 29097191

[2] Sahel J Bonnel S Mrejen S Paques M. Retinitis pigmentosa and other dystrophies. Dev Ophthalmol. (2010) 47:160–7. 10.1159/000320079 20703049

[3] Charbel Issa P Bolz HJ Ebermann I Domeier E Holz FG Scholl HPN. Characterisation of severe rod-cone dystrophy in a consanguineous family with a splice site mutation in the MERTK gene. Br J Ophthalmol. (2009) 93:920–5. 10.1136/bjo.2008.147397 19403518

[4] Abu-Safieh L Alrashed M Anazi S Alkuraya H Khan AO Al-Owain M. Autozygome-guided exome sequencing in retinal dystrophy patients reveals pathogenetic mutations and novel candidate disease genes. Genome Res. (2013) 23:236–47. 10.1101/gr.144105.112 23105016 PMC3561865

[5] Patel N Aldahmesh MA Alkuraya H Anazi S Alsharif H Khan AO. Expanding the clinical, allelic, and locus heterogeneity of retinal dystrophies. Genet. Med. (2016) 18:554–62. 10.1038/gim.2015.127 26355662

[6] Bok D Hall MO. The role of the pigment epithelium in the etiology of inherited retinal dystrophy in the rat. J Cell Biol. (1971) 49:664–82. 10.1083/jcb.49.3.664 5092207 PMC2108484

[7] Zhang JX Wang NL Lu QJ. Development of gene and stem cell therapy for ocular neurodegeneration. Int J Ophthalmol. (2015) 3:622–30. 10.3980/j.issn.2222-3959.2015.03.33 26086019 PMC4458674

[8] Boye SE Boye SL Lewin AS Hauswirth WW. A comprehensive review of retinal gene therapy. Mol Ther. (2013) 21:509–19. 10.1038/mt.2012.280 23358189 PMC3642288

[9] Conlon TJ Deng WT Erger K Cossette T Pang JJ Ryals R. Preclinical potency and safety studies of an AAV_2_-mediated gene therapy vector for the treatment of MERTK associated retinitis pigmentosa. Hum Gene Ther Clin Dev. (2013) 24:23–8. 10.1089/humc.2013.037 23692380 PMC3856558

[10] Ghazi NG Abboud EB Nowilaty SR Alkuraya H Alhommadi A Cai H. Treatment of retinitis pigmentosa due to MERTK mutations by ocular subretinal injection of adeno-associated virus gene vector: results of a phase I trial. Hum Genet. (2016) 135:327–43. 10.1007/s00439-016-1637-y 26825853

[11] Law AL Parinot C Chatagnon J Gravez B Sahel J-A Bhattacharya SS. Cleavage of Mer Tyrosine Kinase (MERTK) from the cell surface contributes to the regulation of retinal phagocytosis. J Biol Chem. (2015) 290:4941–52. 10.1074/jbc.M114.628297 25538233 PMC4335232

[12] Pelaez D. Stem Cells for microenvironmental modulation and retinal regeneration. Curr Tissue Eng. (2016) 5:52–9. 10.2174/2211542004666150713191117

[13] Albert R Kristóf E Zahuczky G Tóth M Veréb Z Oláh B. Triamcinolone regulated apopto-phagocytic gene expression patterns in the clearance of dying retinal pigment epithelial cells. A key role of MERTK in the enhanced phagocytosis. BBA Gen Subjects. (2015) 1850:435–46. 10.1016/j.bbagen.2014.10.026 25450174

[14] Putney SD Burke PA. Improving protein therapeutics with sustained-release formulations. Nat. Biotechnol. (1998) 16:153–7. 10.1038/nbt0298-153 9487521

[15] Rauck BM Novosat TL Oudega M Wang Y. Biocompatibility of a coacervate-based controlled release system for protein delivery to the injured spinal cord. Acta Biomater. (2015) 11:204–11. 10.1016/j.actbio.2014.09.037 25266504 PMC4256151

[16] Li Z Qu T Ding C Ma C Sun H Li S. Injectable gelatin derivative hydrogels with sustained vascular endothelial growth factor release for induced angiogenesis. Acta Biomater. (2015) 13:88–100. 10.1016/j.actbio.2014.11.002 25462840 PMC4293253

[17] Zhang Z Tongchusak S Mizukami Y Kang YJ Ioji T Touma M. Induction of anti-tumor cytotoxic T cell responses through PLGA-nanoparticle mediated antigen delivery. Biomaterials. (2011) 32:3666–78. 10.1016/j.biomaterials.2011.01.067 21345488

[18] Wu C Baldursdottir S Yang M Mu H. Lipid and PLGA hybrid microparticles as carriers for protein delivery. J Drug Deliv Sci Technol. (2018) 43:65–72. 10.1016/j.jddst.2017.09.006

[19] Kurowska-Stolarska M Alivernini S Garcia Melchor E Elmesmari A Tolusso B Tange C. MicroRNA-34a dependent regulation of AXL controls the activation of dendritic cells in inflammatory arthritis. Nat Commun. (2017) 8:1–13. 10.1038/ncomms15877 28639625 PMC5489689

[20] Koch S Sothilingam V Garrido MG Tanimoto N Becirovic E Koch F. Gene therapy restores vision and delays degeneration in the CNGB1^1/1^ mouse model of retinitis pigmentosa. Hum Mol Genet. (2012) 21:4486–96. 10.1093/hmg/dds290 22802073

[21] LaVail MM Yasumura D Matthes MT Yang H Hauswirth WW Deng WT. Gene therapy for MERTK-associated retinal degenerations. Adv Exp Med Biol. (2016) 854:487–93. 10.1007/978-3-319-17121-0_65 26427450 PMC4942279

[22] Wert KJ Davis RJ Sancho-Pelluz J Nishina PM Tsang SH. Gene therapy provides long-term visual function in a pre-clinical model of retinitis pigmentosa. Hum Mol Genet. (2013) 22:558–67. 10.1093/hmg/dds466 23108158 PMC3542865

[23] Deng WT Dinculescu A Li Q Boye SL Li J Gorbatyuk MS. Tyrosine-mutant AAV8 delivery of human MERTK provides long-term retinal preservation in RCS rats. Invest Ophthalmol Vis Sci. (2012) 53:1895–904. 10.1167/iovs.11-8831 22408006 PMC3995567

[24] Csaba N Sánchez A Alonso MJ. PLGA: Poloxamer and PLGA: Poloxamine blend nanostructures as carriers for nasal gene delivery. J Control Release. (2006) 113:164–72. 10.1016/j.jconrel.2006.03.017 16759732

[25] Lemke G. Biology of the TAM receptors. Cold Spring Harb Perspect Biol. (2013) 5:a009076. 10.1101/cshperspect.a009076 24186067 PMC3809585

[26] Liao JL Yu J Huang K Hu J Diemer T Ma Z. Molecular signature of primary retinal pigment epithelium and stem-cell-derived RPE cells. Hum Mol Genet. (2010) 19:4229–38. 10.1093/hmg/ddq341 20709808 PMC3115666

