# Supplementary material for: Sustained Release of Gas6 via mPEG-PLGA Nanoparticles Enhances the Therapeutic Effects of MERTK Gene Therapy in RCS Rats
Source: Front Med (Lausanne). 2021 Dec 14;8:794299. doi: 10.3389/fmed.2021.794299 (PMC8712650; doi:10.3389/fmed.2021.794299)
Supplement: Supplementary file 1 [file Presentation_1.pdf]

Figure S1. A-wave of dark-adapted (scotopic) ERG data of the representative RCS rats at 12 dpi and 19 dpi. (A) Scotopic ERG response (12dpi) at a series of intensity from 0.01 to 30 cd*s/m2. Control: n=7; MerTK: n=9; MerTK/Gas6: n=7; MerTK/Gas6 NPs: n=6. (B) Scotopic ERG response (19dpi) at a series of intensity from 0.01 to 30 cd*s/m2. Control: n=6; MerTK: n=5; MerTK/Gas6: n=3; MerTK/Gas6 NPs: n=4. * P<0.05, ** P<0.005.

At 12dpi, the hMERTK/Gas6 NPs group exhibited significantly higher a-wave (68.74 ± 19.32 µV) in dark-adapted ERG responses at 0.3 cd.s/m2 than other groups. The a-wave amplitudes of the hMERTK group (50.88 ± 9.23 µV) and hMERTK/Gas6 group (46.73 ± 14.84 µV) were also higher than that of the control group (27.39 ± 4.74 µV) at 0.3 cd.s/m2; and there is no significant difference between the hMERTK group and hMERTK/Gas6 group. At 19dpi, the a-wave of dark-adapted ERG responses in the hMERTK/Gas6 NPs group(71.47 ± 38.44 µV) was significantly higher than that of the other groups at 3 cd.s/m2. However, there was no significant difference among the hMERTK, hMERTK/Gas6 and control groups at all intensity of stimulus.

Figure S2. The thickness of the outer nuclear layer of the retina. The data are expressed as mean ± SD. * P < 0.05 vs. control group; # P < 0.05 vs. hMERTK/Gas6 NPs group.

The thickness of ONL in control group (14.33 ± 1.14µm) was significantly less than that of the hMERTK (27.17 ± 3.12µm), hMERTK/Gas6 (26.00 ± 2.61µm), and hMERTK/Gas6 NPs group (35.14 ± 3.68µm). A statistically significant difference was observed between the hMERTK/Gas6 NP group and the other 2 treatment groups. However, there was no significant difference between the hMERTK and hMERTK/Gas6 group.

Figure S3. Map of pAAV2-BEST1-hMERTK and pAAV2-BEST1-EGFP plasmid
