# Supplementary material for: Sustained Release of Gas6 via mPEG-PLGA Nanoparticles Enhances the Therapeutic Effects of MERTK Gene Therapy in RCS Rats
Source: Front Med (Lausanne). 2021 Dec 14;8:794299. doi: 10.3389/fmed.2021.794299 (PMC8712650; doi:10.3389/fmed.2021.794299)
Supplement: Supplementary file 2 [file Table_1.docx]

Table S1. Sequence of *BEST1* promoter

|  | Base sequence | | | | |
| --- | --- | --- | --- | --- | --- |
| 1 | aattctgtca | ttttactagg | gtgatgaaat | tcccaagcaa | caccatcctt |
| 51 | ttcagataag | ggcactgagg | ctgagagagg | agctgaaacc | tacccggggt |
| 101 | caccacacac | aggtggcaag | gctgggacca | gaaaccagga | ctgttgactg |
| 151 | cagcccggta | ttcattcttt | ccatagccca | cagggctgtc | aaagacccca |
| 201 | gggcctagtc | agaggctcct | ccttcctgga | gagttcctgg | cacagaagtt |
| 251 | gaagctcagc | acagccccct | aacccccaac | tctctctgca | aggcctcagg |
| 301 | ggtcagaaca | ctggtggagc | agatccttta | gcctctggat | tttagggcca |
| 351 | tggtagaggg | ggtgttgccc | taaattccag | ccctggtctc | agcccaacac |
| 401 | cctccaagaa | gaaattagag | gggccatggc | caggctgtgc | tagccgttgc |
| 451 | ttctgagcag | attacaagaa | gggactaaga | caaggactcc | tttgtggagg |
| 501 | tcctggctta | gggagtcaag | tgacggcggc | tcagcactca | cgtgggcagt |
| 551 | gccagcctct | aagagtgggc | aggggcactg | gccacagagt | cccagggagt |
| 601 | cccaccagcc | tagtcgccag | acc |  |  |
