# Supplementary figures and images for: Sustained Release of Gas6 via mPEG-PLGA Nanoparticles Enhances the Therapeutic Effects of MERTK Gene Therapy in RCS Rats
Source: Front Med (Lausanne). 2021 Dec 14;8:794299. doi: 10.3389/fmed.2021.794299 (PMC8712650; doi:10.3389/fmed.2021.794299)

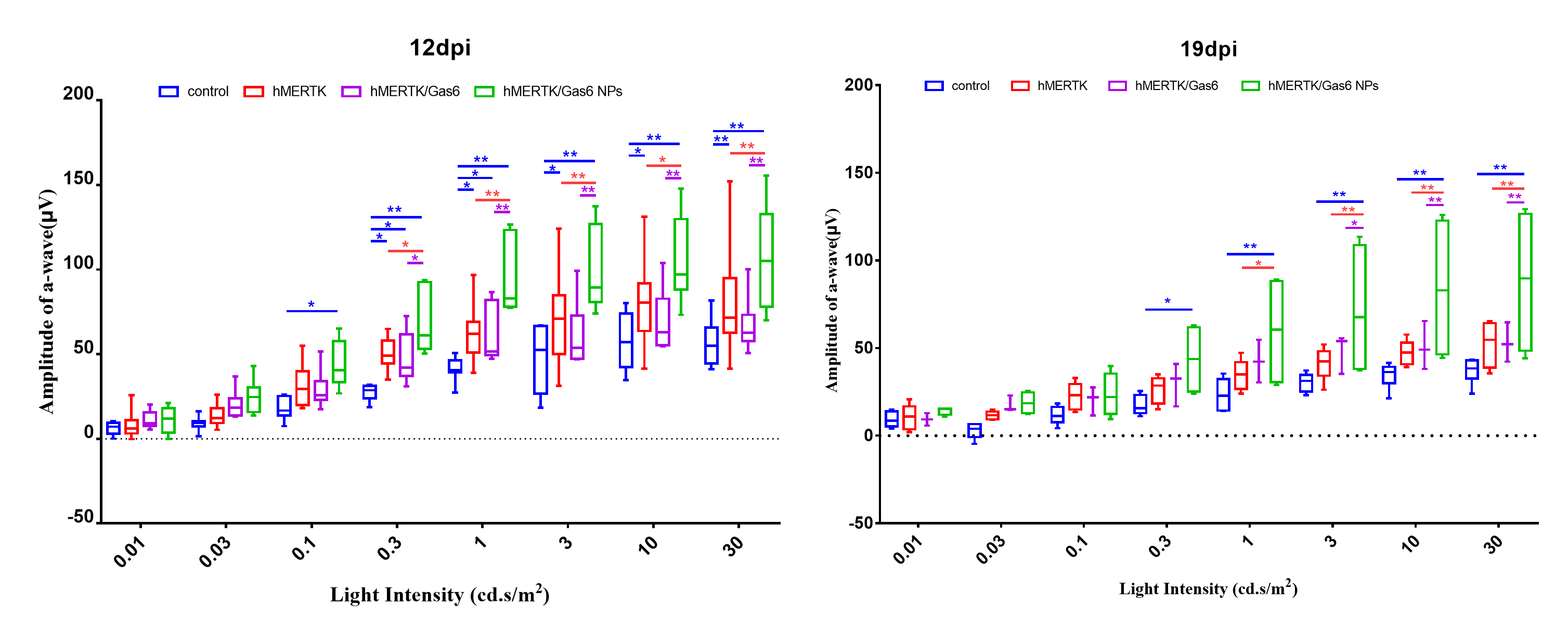

Supplement: Supplementary file 3 [file Image_1.tif]

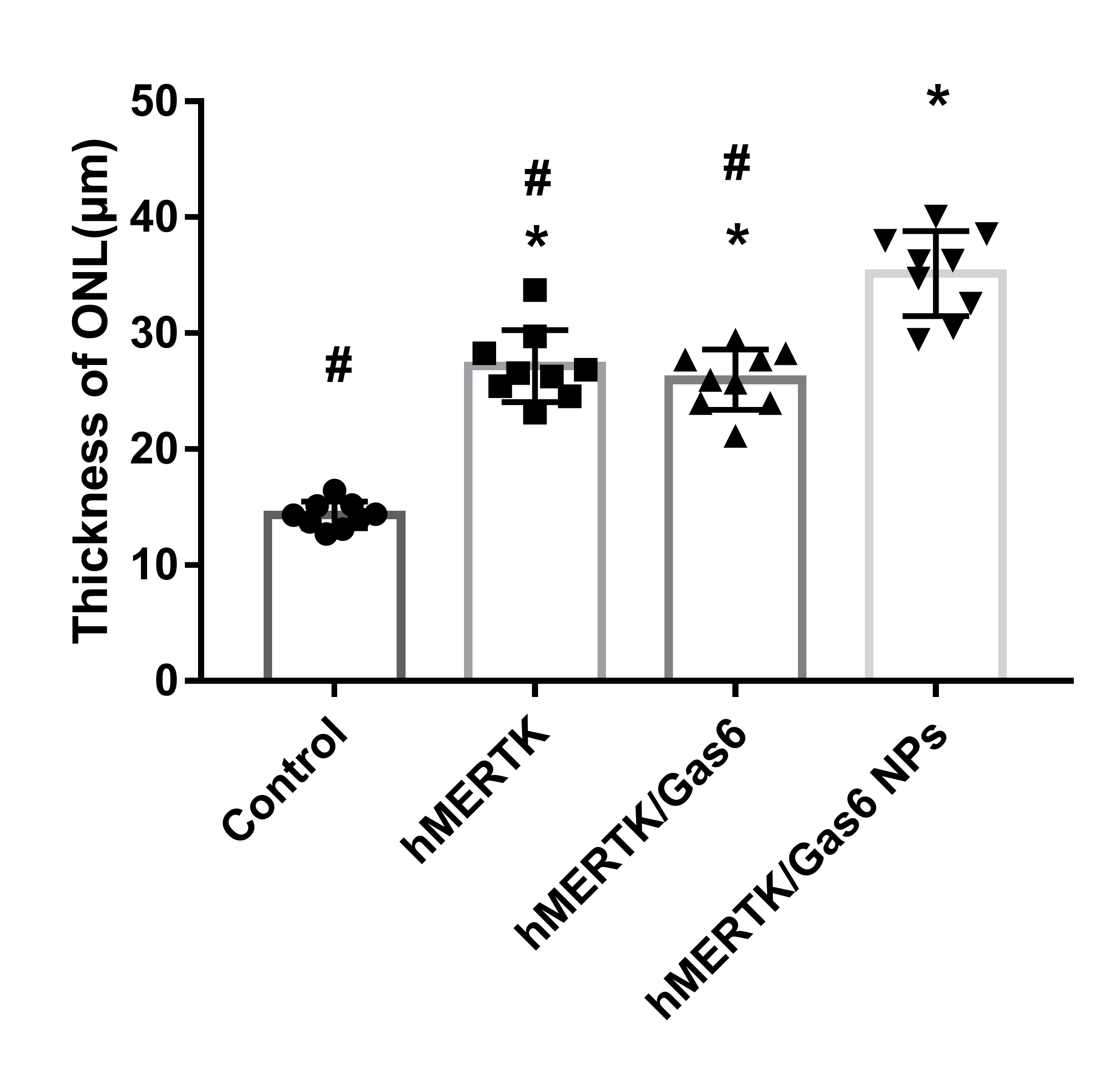

Supplement: Supplementary file 4 [file Image_2.tif]

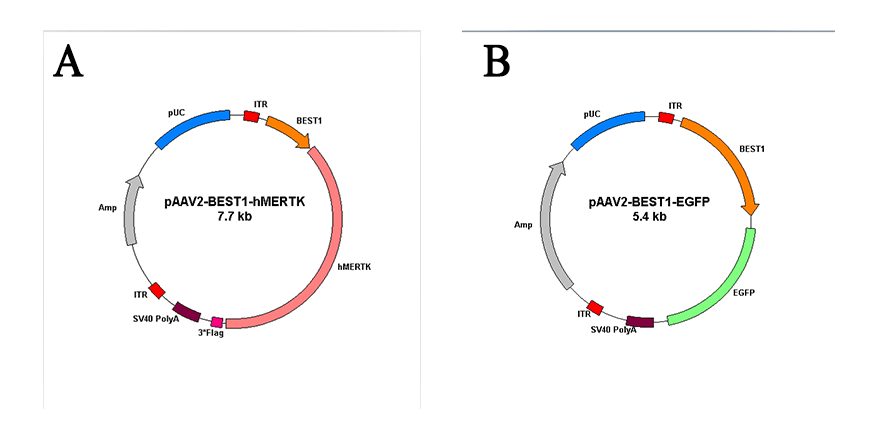

Supplement: Supplementary file 5 [file Image_3.tif]
